# Development of ε-poly(L-lysine) carbon dots-modified magnetic nanoparticles and their applications as novel antibacterial agents

**DOI:** 10.3389/fchem.2023.1184592

**Published:** 2023-04-07

**Authors:** Yuying Jiang, Xinkai Xu, Jinglin Lu, Chuqiang Yin, Guotai Li, Longjian Bai, Tiantian Zhang, Jianning Mo, Xiaoyu Wang, Qiang Shi, Ting Wang, Qihui Zhou

**Affiliations:** ^1^ The Affliated Hospital of Qingdao University, Qingdao University, Qingdao, China; ^2^ School of Stomatology, Qingdao University, Qingdao, China; ^3^ School of Rehabilitation Sciences and Engineering, University of Health and Rehabilitation Sciences, Qingdao, China; ^4^ Moji-Nano Technology Co. Ltd, Yantai, China; ^5^ Zhejiang Engineering Research Center for Tissue Repair Materials, Wenzhou Institute, University of Chinese Academy of Sciences, Wenzhou, China

**Keywords:** magnetic nanoparticles, carbon dots, antibacteria, smart nanomaterials, cytocompatibility

## Abstract

Magnetic nanoparticles (MNPs) are widely applied in antibacterial therapy owing to their distinct nanoscale structure, intrinsic peroxidase-like activities, and magnetic behavior. However, some deficiencies, such as the tendency to aggregate in water, unsatisfactory biocompatibility, and limited antibacterial effect, hindered their further clinical applications. Surface modification of MNPs is one of the main strategies to improve their (bio)physicochemical properties and enhance biological functions. Herein, antibacterial ε-poly (L-lysine) carbon dots (PL-CDs) modified MNPs (CMNPs) were synthesized to investigate their performance in eliminating pathogenic bacteria. It was found that the PL-CDs were successfully loaded on the surface of MNPs by detecting their morphology, surface charges, functional groups, and other physicochemical properties. The positively charged CMNPs show superparamagnetic properties and are well dispersed in water. Furthermore, bacterial experiments indicate that the CMNPs exhibited highly effective antimicrobial properties against *Staphylococcus aureus*. Notably, the *in vitro* cellular assays show that CMNPs have favorable cytocompatibility. Thus, CMNPs acting as novel smart nanomaterials could offer great potential for the clinical treatment of bacterial infections.

## Graphical Abstract

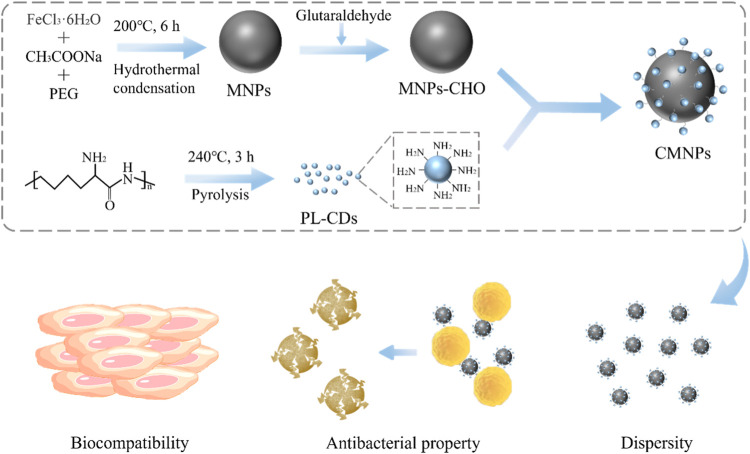

## 1 Introduction

In recent years, nanomaterials have gradually attracted great interest due to their unique characteristics, including small size, easy modification, and high reactivity (Biju, 2014; Zhou et al., 2018b; Wu et al., 2020; Tang et al., 2021; Zheng et al., 2021). Particularly, they have been widely applied in biomedical fields, such as tissue engineering, bioimaging, cancer therapy, and antibacterial applications (Ku et al., 2013; Zhou et al., 2016; Liguori et al., 2019; Tang X. et al., 2020; Makabenta et al., 2021; Wang et al., 2021; Huang et al., 2022; Song et al., 2022). Among various nanomaterials, magnetic nanoparticles (MNPs), which have been approved by the U.S. Food and Drug Administration (FDA), received considerable attention for their broad biomedical applications (e.g., magnetic resonance imagining, diagnostics, drug delivery, hyperthermia, etc.) (Arias et al., 2018; Vangijzegem et al., 2019; Tang C. et al., 2020). Owing to their distinctive characteristics, including superparamagnetic behavior, peroxidase-like activity, small particle size, and diverse surface functionalization, they have promising applications in antibacterial therapy (Dong et al., 2021; Ji et al., 2021). Additionally, the response of MNPs to external magnetic fields made them stand out and offered broad prospects for the development of smart antibacterial nanomaterials (Hong et al., 2021). In this topic, MNPs-based nanomaterials have been used to target and eliminate biofilms in highly confined anatomical surfaces in the interior of human teeth (Hwang et al., 2019).

Although previous studies have demonstrated that MNPs could combat various pathogenic bacteria, the tendency of MNPs to aggregate in water may limit their antibacterial effects (Ahamed et al., 2011; de Toledo et al., 2018; Allafchian and Hosseini, 2019). Moreover, research indicated that their antibacterial ability still needs to be improved. Besides, the intrinsic toxicity of nanoparticles and the possibility of causing an immune response leads to unsatisfactory biocompatibility of MNPs (Lin Y. et al., 2020). Therefore, developing a surface modification strategy based on MNPs is required.

Carbon-based nanomaterials (CBNs) (e.g., fullerene, graphene, carbon nanotubes, etc.) are widely applied in biomedical fields for their excellent biosafety and unique physicochemical properties (Li et al., 2021; Cui et al., 2022). Among these, as a new star of the carbon family, carbon dots (CDs) have attracted increasing attention since their first discovery during the purification of single-walled carbon nanotubes (SWCNTs) in 2004 (Xu et al., 2004). CDs are generally defined as 0-dimensional carbon nanomaterials with sizes below 10 nm and instinct fluorescence properties (He et al., 2022). They have attracted wide attention for their superior fluorescence characteristic, and they are frequently used as the fluorescence imaging component in composite materials (Lin C. et al., 2020). In addition to optical characteristics, CDs have also been reported to exhibit excellent antibacterial properties and biosafety (Dou et al., 2015; Li J. et al., 2020). As an emerging material with both fluorescence imaging and antibacterial capabilities, CDs are ideal candidates for constructing multifunctional composite intelligent nanomaterials.

CDs have broad prospects in antibacterial applications known for their distinctive characteristics, such as high biocompatibility, favorable dispersibility, ultrasmall size, modifiable surface, and unique optical properties (Fan et al., 2014; Liu et al., 2020; Varghese and Balachandran, 2021; Tang et al., 2022). It was demonstrated that CDs could exhibit efficient antibacterial properties through mechanisms, such as electronic interactions, retaining functional groups of the precursors, and reactive oxygen species (ROS) generation (Sun et al., 2021; Tang et al., 2022; Du et al., 2023). The multiple antibacterial mechanisms are conducive for CDs to eradicate pathogenic bacteria with high efficiency. Moreover, the abundant functional groups on the surface of CDs enable them to combine with other materials and enhance their antibacterial functions. For instance, Yang *et al.* combined antibacterial ε-poly (L-lysine) CDs (PL-CDs) and oxidized dextran (ODA) through Schiff-base to construct an injectable, self-healing PL-CD@ODA hydrogel, the antibacterial efficiency of which against 10^7^ CFU/mL of *S. aureus* (*Staphylococcus aureus*) reaches nearly 100% (Yang et al., 2021). In addition, research indicates that modifying CDs on inorganic nanomaterial could increase dispersity, thereby broadening their applications under physiological conditions (Jiao et al., 2016). It can be assumed that CDs are ideal materials for the surface modification of MNPs. Their favorable biocompatibility would reduce the intrinsic toxicity of MNPs, and they could improve the dispersion of MNPs as well as exhibit excellent antibacterial ability.

Inspired by the introduction above, we hypothesize that PL-CDs-modified MNPs (CMNPs) could exhibit favorable bio-physicochemical properties to eliminate pathogenic bacteria, which has yet to be reported. To test the hypothesis, CMNPs were obtained by linking PL-CDs and MNPs through the imine bond generated by the Schiff-base reaction. The physicochemical features of CMNPs (i.e., morphology, functional groups, crystalline structure, dispersity, surface potential, and magnetic properties) were systematically measured by different characterization techniques. The antibacterial effects of CMNPs against *S. aureus* were demonstrated *in vitro*. Further, we investigated the cytocompatibility and hemocompatibility of CMNPs through cellular and hemolysis assays.

## 2 Experimental section

### 2.1 Materials

Sodium acetate (NaOAc), FeCl_3_.6H_2_O, ethylene glycol, and polyethylene glycol (Mw = 6,000 Da) (PEG) were purchased from Sinopharm Chemical Reagent Co. Ltd. ε-poly (L-lysine) hydrochloride (Mw ∼ 4,000) was purchased by Shanghai D&B Biological Science and Technology Co. Ltd. Tryptone Soy Broth (TSB), tetramethylrhodamine isothiocyanate (TRITC)-Phalloidin, agar, and 4′6-diamidino-2-phenylindole (DAPI) were offered from Solarbio Science & Technology Co. Ltd. (Beijing, China). Cell Counting Kit-8 (CCK-8) was offered by MedChemExpress Co. Ltd. (Shanghai, China). Dulbecco’s modified Eagle’s medium (DMEM), fetal bovine serum (FBS), streptomycin, and penicillin were provided by Biological Industries Ltd. (Beit-Haemek, Israel). All other materials were of analytic level and used without further refinement.

### 2.2 Preparation and characterization of PL-CDs, MNPs and CMNPs

#### 2.2.1 Preparation of PL-CDs by the top-down approach

ε-poly (L-lysine) hydrochloride (1 g) was taken in the crucible. The crucible was put in an oven, heated to 240°C at a rate of 4°C/min and kept for 3 h, and then cooled naturally down to room temperature (RT). The brownish-black residue was ground into a very fine powder in a mortar and dissolved in 20 ml of double distilled water (ddH_2_O). The intermixture was sonicated in a beaker for 30 min, then centrifuged (10,000 rad/min) for 60 min. The supernatant was dialyzed (MWCO = 5,000 Da) and lyophilized to obtain yellow-brownish flocculent product.

#### 2.2.2 Preparation of Fe_3_O_4_ MNPs

Fe_3_O_4_ magnetic nanoclusters were prepared by hydrothermal method. FeCl_3_.6H_2_O (1.25 g) was dissolved in ethylene glycol (40 ml) to form a light orange solution, then NaOAc (3.6 g) and PEG (1 g) were added and stirred vigorously until the mixture became brown rapidly. After 30 min of shaking, the reaction solution was put into the 50 ml stainless-steel autoclave with polytetrafluoroethylene liner and heated at 200°C for 6 h. After heating, that reaction solution was cooled to RT, and the black product was washed three times sequentially with ddH_2_O and ethanol. The final product was lyophilized in a lyophilizer to obtain MNPs.

#### 2.2.3 Preparation of CMNPs

First, MNPs (200 mg) were dispersed in ethanol (50 ml) and sonicated for 10 min for activation. 10% glutaraldehyde (20 ml) was added to the mixed solution and let stand at RT for 2 h. Then, the material was washed three times with ddH_2_O and stored overnight at 4°C in phosphate-buffered saline (PBS, pH = 7.4, 0.1 M). After that, the lyophilized PL-CDs were dissolved in PBS (pH = 6.0, 0.01 M, 50 ml) to form a 1 mg/ml solution. MNPs collected with an activated magnet were added into the solution subsequently. The mixed solution was shaken at 100 rad/min for 24 h at RT. Finally, the material was gathered with a magnet, washed three times, and lyophilized to obtain the target material CMNPs.

#### 2.2.4 Physicochemical characterization

The shape and size of the samples were observed with a transmission electron microscope (TEM, Mic JEM-1200EX, Japan). The particle size distribution was determined by measuring 100 particles in different samples. The particles were selected based on TEM images randomly.

Ultraviolet-Visible (UV-Vis) spectra were obtained using a UV spectrophotometer (UV-8000, Shanghai Midas Instrument Co., Ltd., China).

PL-CDs fluorescence spectra were performed on a FLS980 fluorescence spectrometer (Edinburgh Instruments Ltd., United Kingdom) with emission spectra recorded between 300 and 460 nm.

Samples were analyzed using a Nicolet iN10 Fourier transform infrared (FT-IR) spectrometer (Thermo Fisher Scientific, Waltham, MA, United States) in the range of 500–4,000 cm^−1^, with a scan resolution of 2 cm^−1^ for FT-IR spectra, with 32 scans.

XRD spectra were performed by Ultima IV (Rigaku Corporation, Japan) to confirm the crystal structure. Under the Cu-Kα radiation conditions of voltage 40 kV and current 30 mA (*λ* = 1.5418 Å), the scanning rate was 0.03°(2θ)/min, and the samples were examined between 5° and 50° (2θ).

The zeta potential Z) of the nanomaterials was tested using dynamic light scattering on the Zetasizer Nano ZSE. The MNPs and CMNPs samples were tested at the same concentration and were dispersed in ddH_2_O.

Magnetic measurement was performed at RT *via* a vibrating sample magnetometer (PPMS-9, Quantum Design, United States). The system field strength of the longitudinal magnets was ±9 T.

### 2.3 Bacterial experiment

#### 2.3.1 Bacterial culture

*Staphylococcus aureus* was grown and incubated at 37°C. Colonies were isolated from TSB agar plates, suspended in TSB broth, and grew at 37°C overnight. *Staphylococcus aureus* was collected by centrifugation during the exponential growth phase. The denseness of that bacterial suspension was normalized to a McFarlane standard of 0.5, and the number of colony-forming units (CFU) was approximately 10^6^ CFU/ml.

#### 2.3.2 Antibacterial tests

Bacterial suspensions in untreated TSB medium were added to the control group. To obtain distinct concentrations of 0.1, 0.5, 1.0, and 2.0 mg/ml, MNPs and CMNPs were added to 1 ml of TSB medium in a test tube. 1 ml of *S. aureus* 10^6^ CFU/ml suspension was also added to each tube separately. After 24 h of incubation at 37°C, these cultures in each culture tube were diluted, and 20 µl solution from the last diluted culture tube was token and incubated dropwise on a sterile TSB agar plate overnight at 37°C. Each experiment was repeated three times.

### 2.4 Cell cytotoxicity assay

Mouse calvarial osteoblast progenitor cells (MC3T3-E1) were obtained from the Cell Culture Center, Shanghai Institutes for Biological Sciences, Chinese Academy of Sciences.

To examine the effect of MNPs on cell adhesion, cells were co-cultured in different concentrations of MNP-based samples. MNPs and CMNPs samples were added to a basal medium containing DMEM supplemented with 1% penicillin/streptomycin and 10% FBS at different sample concentrations. Cells were seeded at the density of 2×10^4^ cells per well. After culturing for 24 h, 1 ml of the sample was added and incubated with cells for 24 h.

After incubation, the cells were fixed with 4% paraformaldehyde for 20 min at RT, then treated with 0.5% Triton X-100 solution. The nuclei and cytoskeleton were then stained with DAPI and TRITC-phalloidin (Solarbio, China), respectively. Images were recorded using an inverted fluorescence microscope (Nikon A1 MP, Japan). Cell expansion area and elongation were quantified.

Cell viability was detected by CCK-8 (Med Chem Express, Shanghai). Cells were seeded at a density of 2 × 10^4^ cells/well onto 24-well plates for 24 h. After that, MNPs and CMNPs solutions were added and incubated for 12 and 24 h. 10 μl of CCK-8 reagent solution and 100 µl of fresh medium was added to each well of the plate. Then OD_450_ was measured with a microplate reader. Cell viability was calculated using the value expressed as a percentage of control wells. The viability of MC3T3-E1 after MNP-based sample treatment compared to cells exposed to no material treatment was calculated as follows.

$$Relative\;viability\;\left(\%\right)=\frac{{OD}_{Test450}}{{OD}_{Control450}}\times 100\%$$

Experiments were performed in triplicate and repeated three times independently.

### 2.5 Hemolysis assay

Fresh eye blood of standard deviation (SD) healthy rats was collected, centrifuged at 5,000 rpm for 5 min, and washed three times with PBS. The blood cells were resuspended in PBS to obtain a final suspension. After that, 0.5 ml of different concentrations of MNPs and CMNPs were compounded with the erythrocyte suspension. After that, the suspension was hatched for 1 h at 37°C, and it was centrifuged at 5,000 rpm for 5 min. Then the absorbance of the supernatant was measured at 540 nm. PBS and ddH_2_O were used as negative ( − ) and positive (+) controls, respectively. The degree of hemolysis (%) was calculated according to the following formula:

$$Hemolysis\;\%=\frac{Abs-Abs\left(-\right)}{Abs\left(+\right)-Abs\left(-\right)}\times 100\%$$

Abs represents the absorbance of the erythrocyte suspension after contacting the substance, and the absorbance values of the erythrocyte suspension utilized as positive and negative control are Abs (+) and Abs ( − ), respectively.

### 2.6 Statistical analysis

All data points were expressed as mean ± standard deviation (SD). Statistical analysis was carried out by GraphPad PRISM 8.0. Differences between groups were checked by one-way analysis of variance (ANOVA) and Tukey’s test. Error bars represent the SD of triplicate measurements per group. Significant difference levels are indicated by different numbers of asterisks, *p < 0.05, **p < 0.01, ***p < 0.001.

## 3 Results and discussion

### 3.1 Preparation and characterization of CMNPs

**FIGURE 1:**
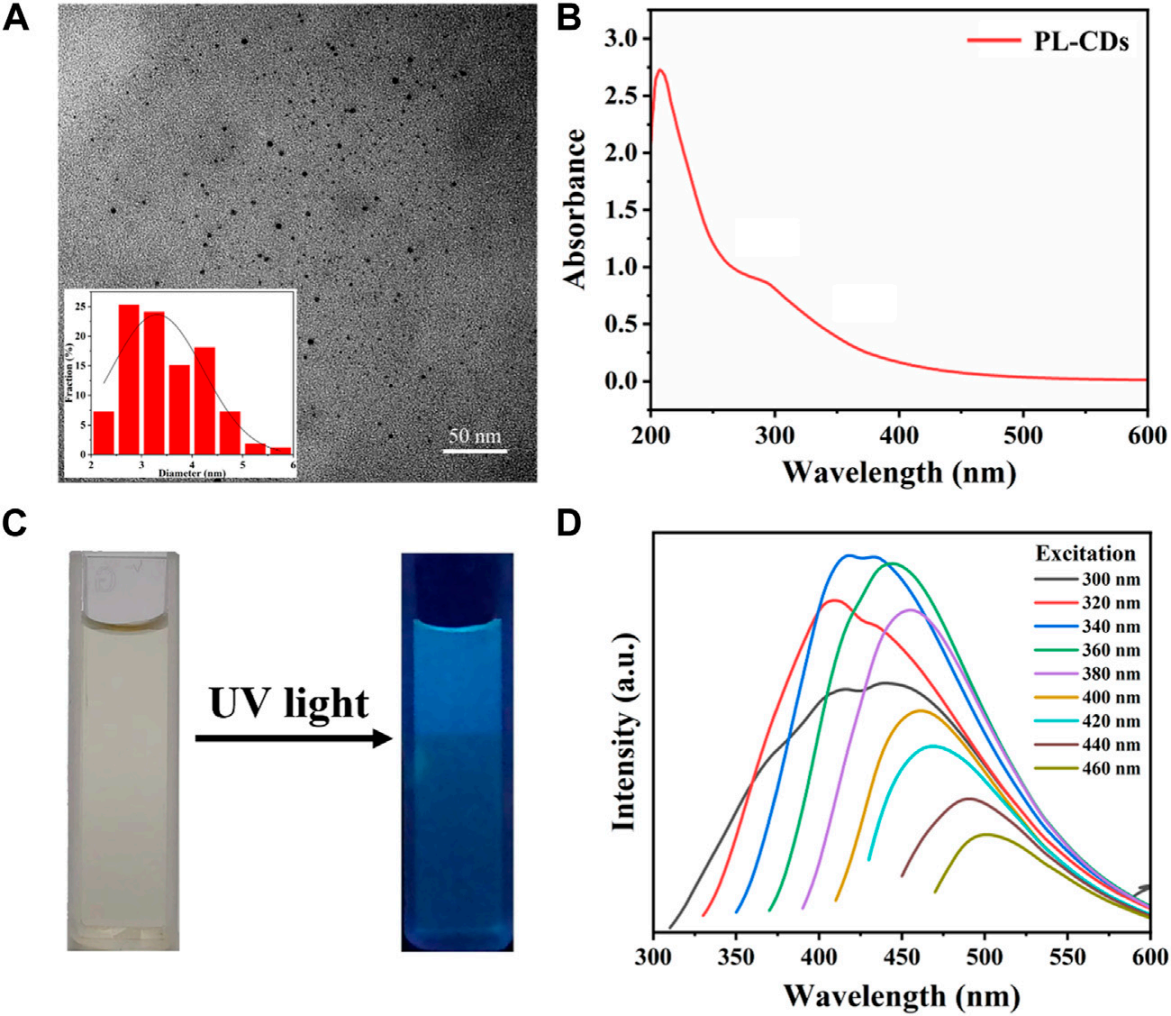
**(A)** TEM image and diameter distribution of PL-CDs. **(B)** UV-vis spectra of PL-CDs. **(C)** Photographs of PL-CDs aqueous solution in sunlight and UV light. **(D)** Photoluminescence of PL-CDs.

Fe_3_O_4_ MNPs were obtained through a modified hydrothermal reduction method between ethylene glycol and FeCl_3_ (Deng et al., 2005). Next, a glutaraldehyde cross-linker was used to modify the surface of MNPs with aldehyde groups for linkage to amino groups in PL-CDs. PL-CDs were synthesized through a one-step pyrolysis method using ε-poly (L-lysine) (PL) as the precursor (Yang et al., 2021). Figure 1A is a TEM image of PL-CDs and shows a spherical structure of monodisperse crystals with a diameter distributed mainly between 2 and 5 nm, and the average diameter was 3.31 ± 0.18 nm. The UV-vis absorption spectrum in Figure 1B indicated two characteristic absorption peaks of soluble PL-CDs. The peak at 270 nm was ascribed to the typical π-π* transition of the aromatic sp^2^ domain (C=C), while the shoulder peak at 300–400 nm corresponded to the n-π* transitions of C=O and C-N bonds (Jian et al., 2017). The results demonstrate that PL-CDs were successfully synthesized.

Further, we investigated the optical properties of the as-prepared PL-CDs. As shown in Figure 1C, the PL-CDs emitted bright blue fluorescence under ultraviolet light irradiation. When the excitation wavelength ranged from 300 to 460 nm, the fluorescence of PL-CDs was stable and excitation wavelength-dependent (Figure 1D). The maximum emission gradually moved in the direction of the long wavelength as the increase of excitation wavelength. This phenomenon could be attributed to the optical selection of different surface traps of PL-CDs at different energies due to their structure inhomogeneity (Liang et al., 2013). Moreover, the shape of the emission spectrum gradually changed as the wavelength of the excitation light increased. In the excitation wavelength of 320–460 nm, a double-peak region (300–340 nm) and a single-peak region (360–460 nm) appeared sequentially. This may be because the emission centers corresponding to the two absorption peaks of PL-CDs in the UV-vis spectrum have different activities at different excitation wavelengths. A double-peak curve indicates that both emission centers were active, while as the excitation wavelength approached the red region of the visible spectrum, only one emission center remained active, and the emission spectrum showed a single peak (Ardekani et al., 2019). The typical optical characteristics mentioned above provided further evidence for the successful preparation of PL-CDs.

**FIGURE 2:**
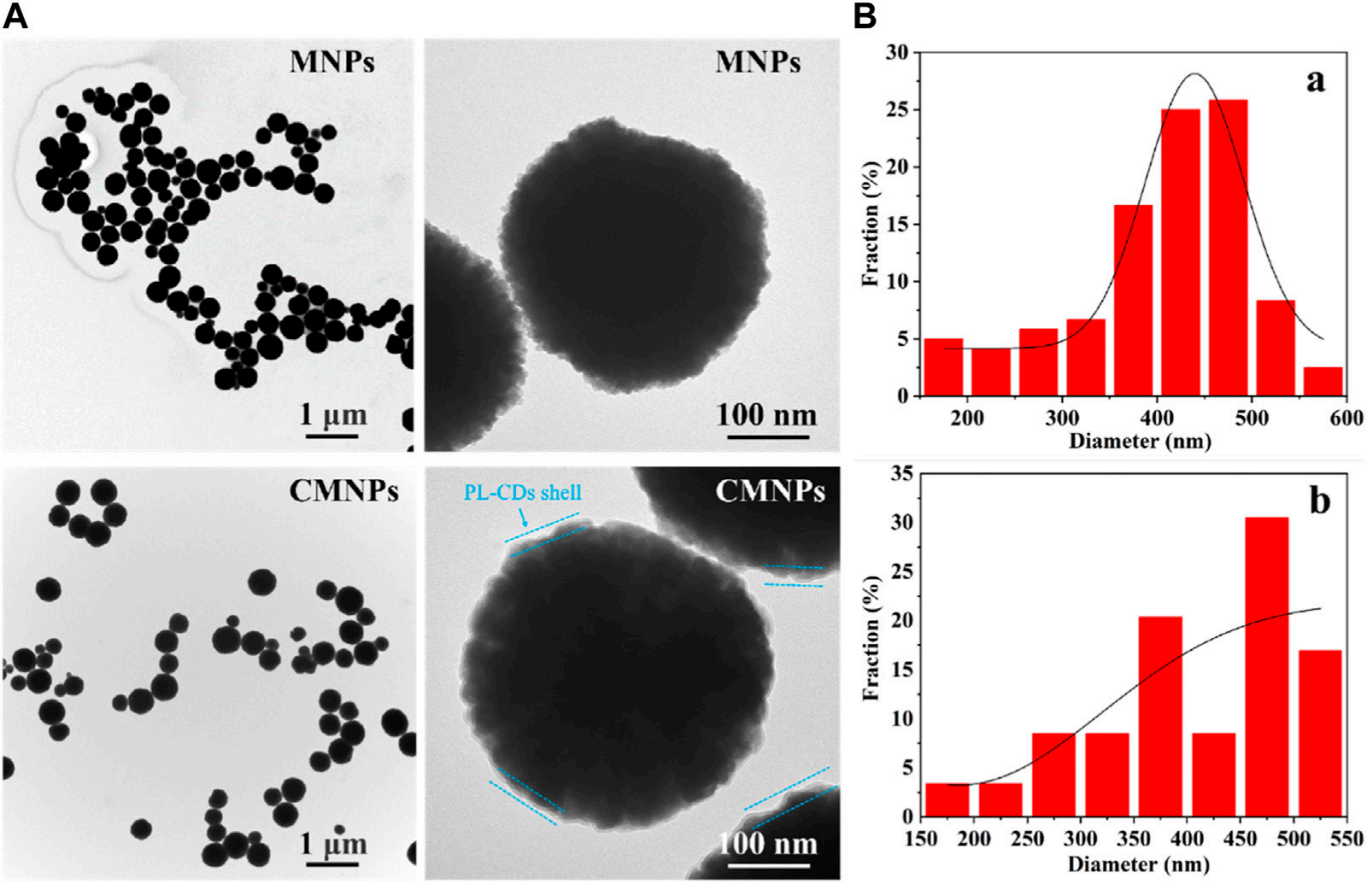
**(A)** TEM images of MNPs and CMNPs. **(B)** Particle size distribution of MNPs (a) and CMNPs (b).

CMNPs were obtained by linking MNPs and PL-CDs through the imine bond generated by the Schiff-base reaction between aldehyde groups modified on the surface of MNPs and the amino groups on the surface of PL-CDs. The combination was black in color and quickly responded to the magnetic field. The morphological characteristics of MNPs and CMNPs were further examined by TEM. Figure 2A shows that both MNPs and CMNPs had a uniform spherical structure, and a thin layer of PL-CDs shell was observed on the surface of CMNPs. The diameter of the shell was 2–5 nm, proving the successful connection of MNPs and PL-CDs. Moreover, the CMNPs in the images were more separate than MNPs, suggesting that they have a better dispersion capacity. Figure 2B shows that the average diameter of CMNPs was 411.82 nm, which was slightly larger than that of MNPs (402.35 nm), probably owing to the PL-CDs loaded on the surface of the MNPs increasing their diameter.

**FIGURE 3:**
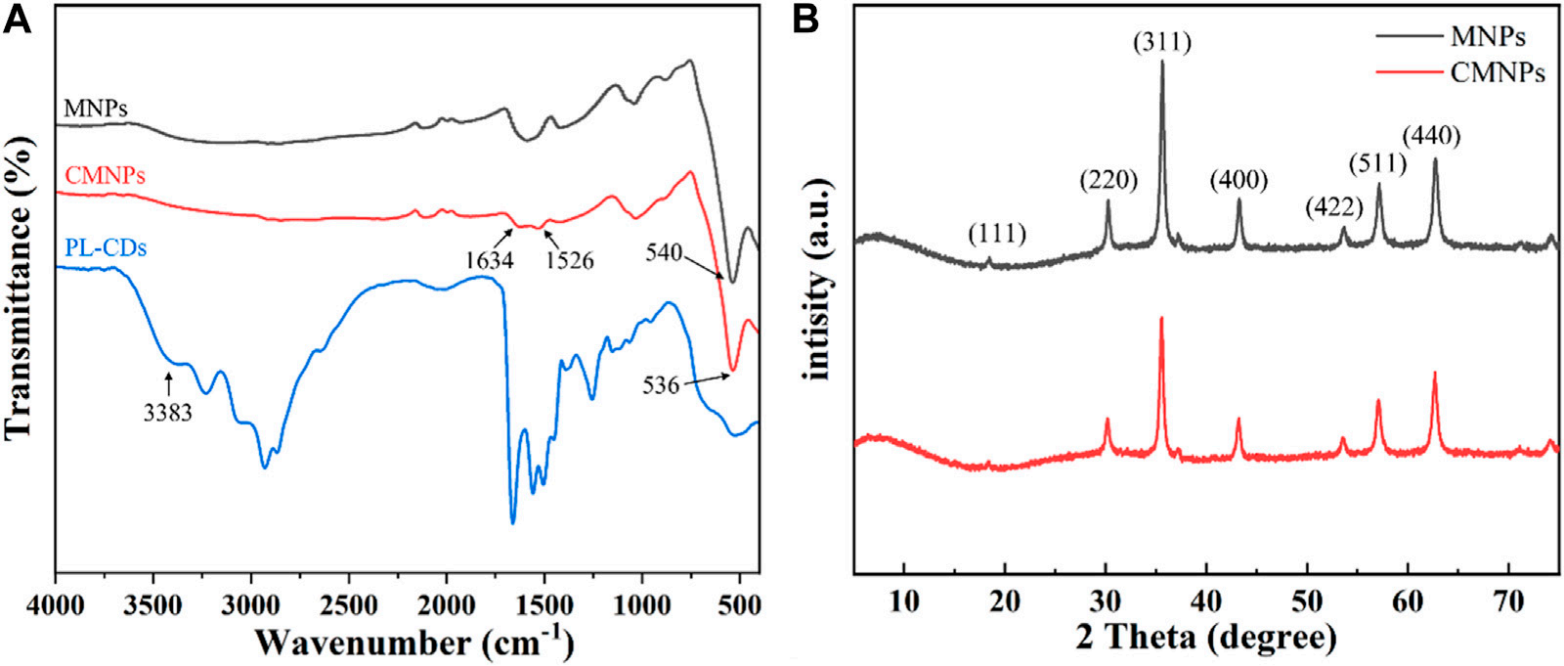
**(A)** FT-IR spectra of MNPs, PL-CDs, and CMNPs. **(B)** XRD patterns of MNPs and CMNPs.

The chemical characteristic of the developed nanomaterials was analyzed by FT-IR. As shown in Figure 3A, the peak at 540 cm^−1^ was a vibrational peak assigned to the Fe-O bond, indicating that MNPs were successfully synthesized and retained during subsequent modification (Deng et al., 2005). For PL-CDs, the peak at 3,383 cm^−1^ was attributed to υNH_2_, while the spectrum of CMNPs showed the peak of υC = N at 1,633 cm^−1^, proving that MNPs and PL-CDs were successfully connected by imine bond (Yang et al., 2021).

To evaluate the effect of the modification of PL-CDs on the crystalline structure and crystallinity of MNPs, XRD analysis on MNPs and CMNPs was performed (Figure 3B). The reflections of 111, 220, 311, 400, 422, 511, and 440 in both MNPs and CMNPs samples were indexed to standard magnetic Fe_3_O_4_ (JCPDS card No. 19–0,629), demonstrating that the MNPs-based samples had an excellent crystal structure and crystallinity of magnetic Fe_3_O_4_ (Deng et al., 2005; El-Desoky et al., 2020). The results indicated that MNPs were successfully synthesized through the modified hydrothermal reduction method and that their crystalline structure remained the same after surface modification of PL-CDs. However, the broad peaks representative of PL-CDs centered at 20°–25° were not detected in CMNPs, possibly due to the poor crystallinity of PL-CDs and their relatively low content in CMNPs (Li et al., 2016; Li et al., 2020c).

**FIGURE 4:**
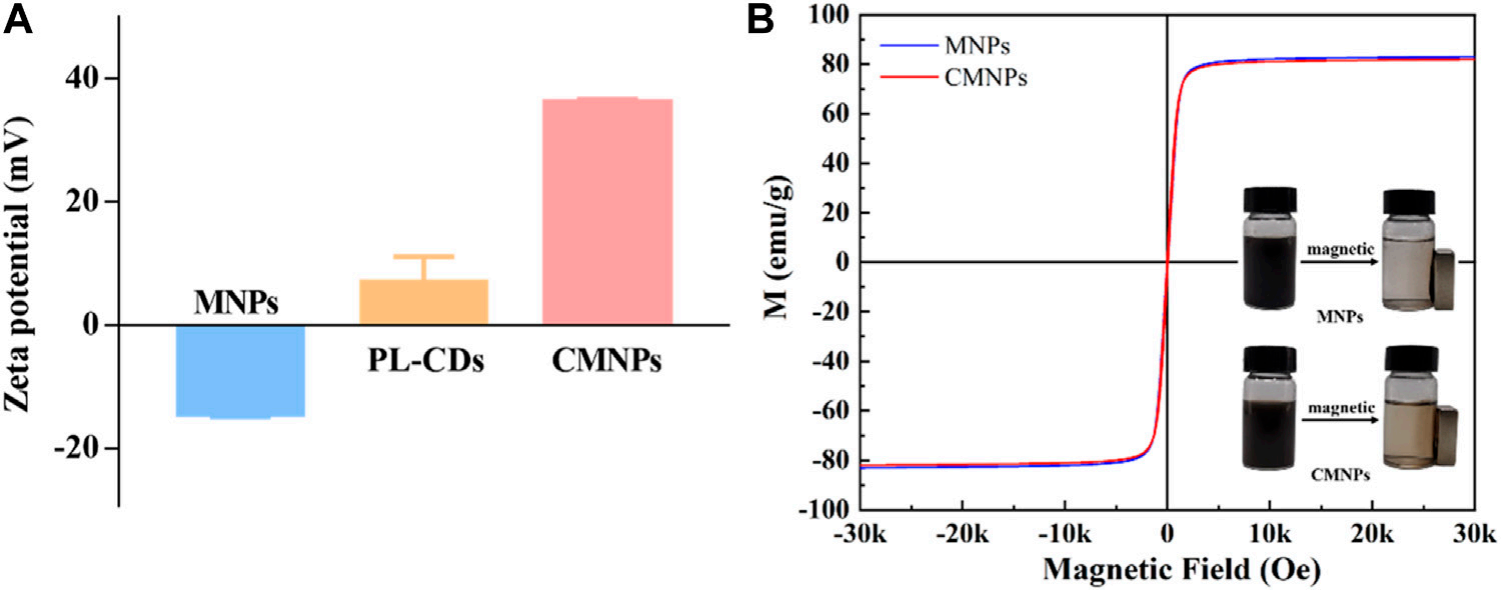
**(A)** Zeta potential of MNPs, PL-CDs, and CMNPs in ddH_2_O. **(B)** Magnetic hysteresis loops of MNPs and CMNPs. The inserted images show that MNPs and CMNPs can be attracted by a magnet.

The zeta potentials of the MNPs, PL-CDs, and CMNPs were separately measured in ddH_2_O. As shown in Figure 4A, the zeta potential of MNPs was negative (−14.23 mV), while the potential of PL-CDs was positive (6.83 mV) owing to a large number of amino groups on their surface. The different charges of MNPs and PL-CDs implied that their electrostatic attraction could potentially promote the synthesis of CMNPs. As we expected, when PL-CDs were modified on the surface of MNPs, the zeta potential of CMNPs significantly increased to 35.97 mV, indicating a successful modification. Interestingly, the potential of CMNPs was higher than PL-CDs. Given that the only factor in correlation with the zeta potential is the surface of a material, we assume that the result may be due to the large number of PL-CDs attached to MNPs (Jastrzebska et al., 2016). Moreover, the absolute value of the zeta potential of CMNPs was also higher than MNPs, illustrating that surface modification of PL-CDs endows CMNPs with better stability in an aqueous solution. The result was consistent with the polydispersity index (PdI) values of the MNP-based samples. The PdI value of MNPs was 0.687. After surface modification, the value decreased to 0.348 for CMNPs, indicating a significant improvement in dispersion capacity.

We further recorded the hysteresis curves of MNPs and CMNPs with a vibrating sample magnetometer. Figure 4B shows that both samples had superparamagnetic properties. Meanwhile, the magnetic saturation of CMNPs (81.96 emu/g) was slightly lower than that of MNPs (83.01 emu/g). The reason could be attributed to the fact that the PL-CDs incorporated on CMNPs belong to non-ferromagnetic material (Dong et al., 2021; Ji et al., 2021).

The inserted images of Figure 4B show that MNPs and CMNPs were collected by a magnet within a short period. It is worth mentioning that the solution of CMNPs after magnetic collection, particularly the area around the magnet, was a little darker than MNPs, indicating that the response of CMNPs to the magnetic field in ddH_2_O was slightly slower than MNPs. The reason for this phenomenon could be attributed to the different hydrodynamic diameters of the two samples. The hydrodynamic diameter of CMNPs in ddH_2_O was smaller than 500 nm, while the diameter of MNPs was larger than 1,000 nm due to the rapid aggregation of the bare MNPs. As reported previously, larger hydrodynamic diameters of nanoparticles in solution are essential for rapid magnetophoretic mobility (Yu et al., 2010). However, in our work, the difference in magnetic responsiveness between the two MNPs-based samples was minimal. The CMNPs exhibited excellent magnetic responsiveness by being responsive to the magnet quickly. The result also reflects that the dispersity of CMNPs was improved after the surface modification of PL-CDs.

It is also worth mentioning that CMNPs were not detected to emit fluorescence under excitation light (300–460 nm). The reason could be that the light adsorption properties of black MNPs mask the fluorescence emitted by PL-CDs (Li J. et al., 2020). The above results collectively demonstrate that PL-CDs were successfully modified on MNPs.

### 3.2 Antibacterial activities of CMNPs

**FIGURE 5:**
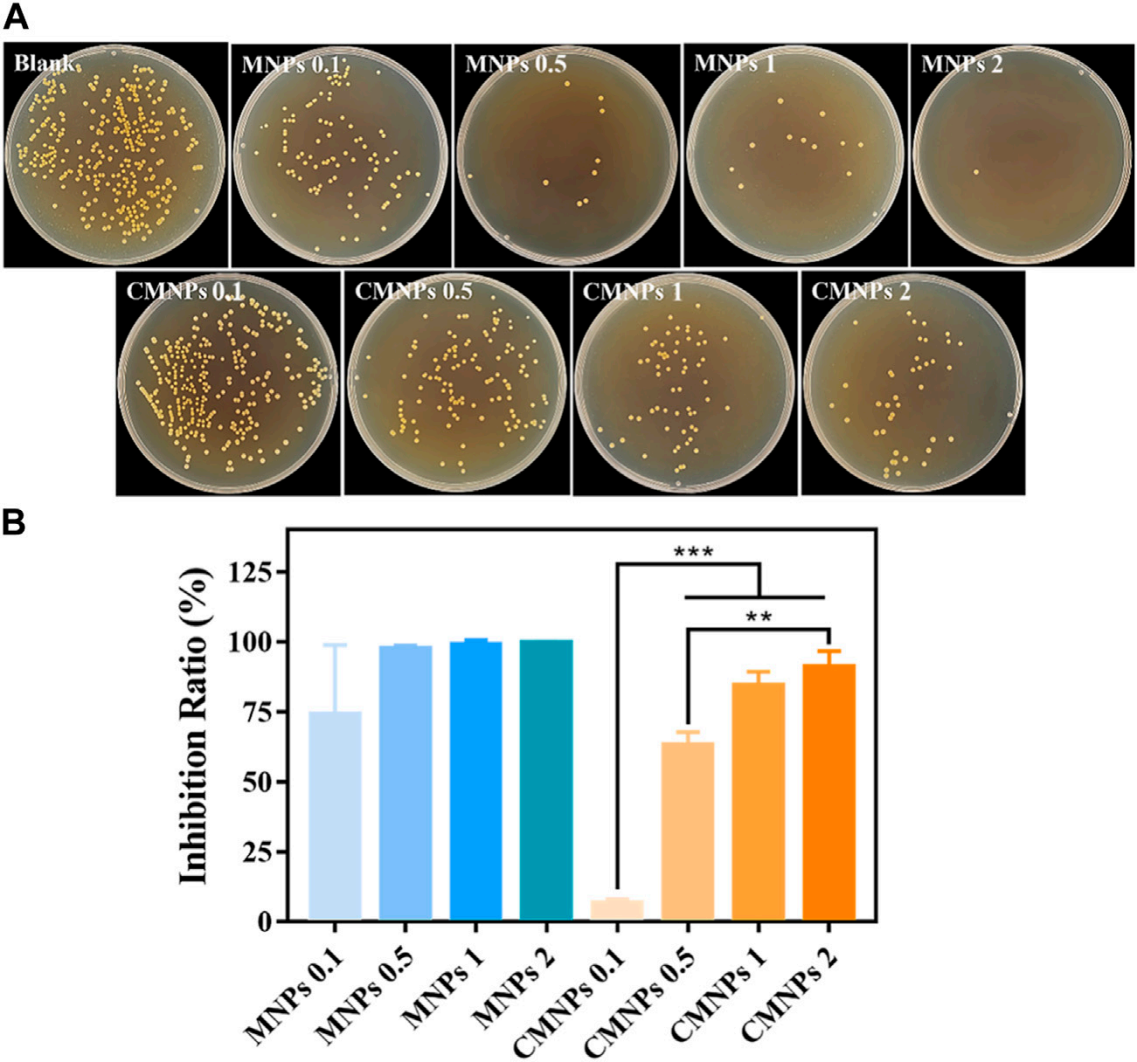
**(A)** Photographs of survived bacterial colonies of *Staphylococcus aureus* treated with different concentrations of MNPs and CMNPs. **(B)** The inhibition ratios of MNPs and CMNPs against *Staphylococcus aureus with* corresponding concentrations.

The CFU count test was employed to investigate the antibacterial activity of MNPs and CMNPs against *S. aureus*. As shown in Figure 5A, the photographs qualitatively demonstrated fewer *S. aureus* bacterial colonies on the MNPs and CMNPs compared with the blank control. Moreover, the colonies in both groups decreased gradually as the concentrations of MNPs and CMNPs increased from 0.1 to 2.0 mg/mL. However, the number of *S. aureus* colonies on the CMNPs was higher at the same material concentrations than MNPs.

The antibacterial efficiency of MNPs and CMNPs against bacteria was calculated for a more intuitive comparison. As shown in Figure 5B, the inhibition ratio of CMNPs was concentration-dependent. CMNPs with high concentrations (i.e.*,*1 and 2 mg/mL) exhibited an inhibition ratio of more than 80%. Particularly, CMNPs at a dosage of 2 mg/ml were able to destroy 91.10% of *S. aureus*, indicating that CMNPs possess the excellent antibacterial capability. However, the inhibition ratio of CMNPs was generally lower than that of MNPs at the same doses, demonstrating that the antibacterial effect of CMNPs was not as efficient as the MNPs group.

Previous studies have reported that cationic CDs could interact with the negatively charged bacterial cell membrane through electrostatic interaction (Li et al., 2020b). CDs with highly positive charge could cause the destruction of the bacterial cell membrane and leakage of the cellular content, thereby resulting in bacterial cell death (Yang et al., 2021). In addition, MNPs possess intrinsic peroxidase-like activity, which enables them to kill bacteria by generating toxic free hydroxyl radicals (Dong et al., 2021). Therefore, we speculate that the antibacterial effect of CMNPs was attributed to the highly positive charge on the surface and the formation of ROS induced by MNPs.

However, the PL-CDs shell on the surface of CMNPs may hinder the contact between MNPs and endogenous H_2_O_2_ in bacteria, thus reducing the catalytic reactions. Accumulation of PL-CDs could be another reason leading to suboptimal antibacterial outcomes. Studies have indicated that particle size is also vital for determining the antibacterial effect of cationic CDs in addition to electrostatic interactions (Sun et al., 2021; Du et al., 2023). Sun et al. investigated the antibacterial activities of cationic CDs with different sizes, and the result indicated that smaller CDs significantly increased bacterial plasma membrane damage, thus remarkably improving the antibacterial ability (Sun et al., 2021). In our work, although the integrated PL-CDs shell on the surface of CMNPs endows the material with high electro-positivity, the shell loses the antibacterial advantage of dispersed CDs with ultra-small particle size, thus reducing antibacterial capacity of PL-CDs. Consequently, although PL-CDs and MNPs have excellent antibacterial activities individually, their combination could not further improve the antibacterial effect of the composite material.

### 3.3 Cyto/hemocompatibility assessment

**FIGURE 6:**
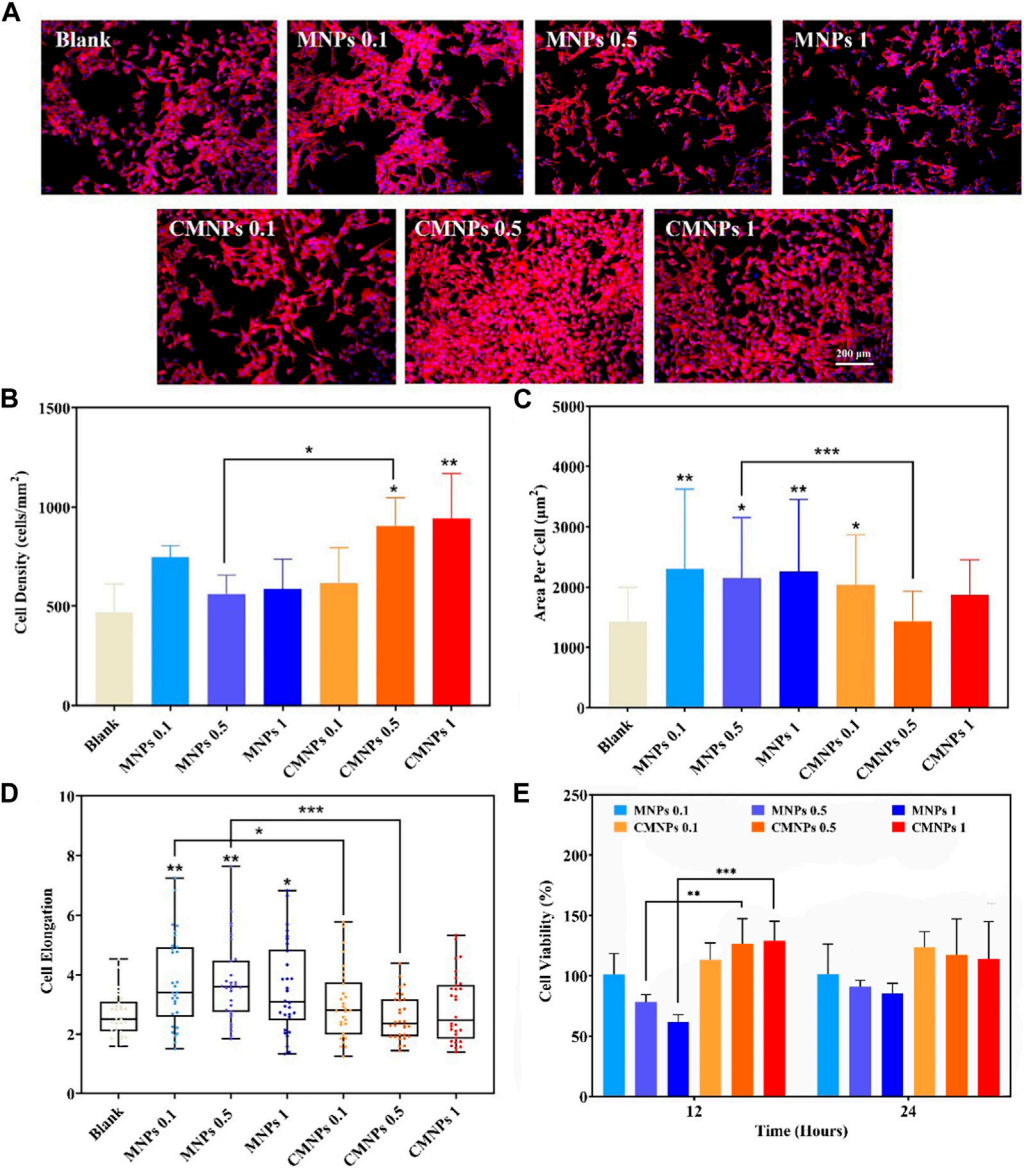
**(A)** Fluorescent images of actin filaments and nuclei staining after co-cultured for 24 h **(B–D)** MC3T3-E1 cell Density, area per cell, and cell elongation after co-cultured for 24 h **(E)** MC3T3-E1 cell viability after co-cultured with MNPs and CMNPs.

The biocompatibility of nanomaterials determines whether it can be applied in the field of biomedicine (Zhou et al., 2018a; Ji et al., 2021; Yan et al., 2022). We co-cultured the murine osteoblastic cell line (MC3T3-E1) with different concentrations of MNPs and CMNPs to evaluate their effects on cell morphology changes, adhesion, and cell viability. The result of such evaluation is prominent in dictating cell viability, proliferation, differentiation, and migration (Tang et al., 2022). As shown in Figure 6A, actin filaments are visible in red using TRITC-phalloidin labeling, and nuclei are labeled blue using DAPI. Each group of MC3T3-E1 cells displayed a typical spindle-like morphology. Whether nanomaterials inhibit the growth and proliferation of cells is one of the critical factors in determining if they have cytotoxicity or not (Li et al., 2020c). We quantitatively analyzed the cells after co-cultured them with the prepared nanomaterial. Figure 6B shows that the cell densities in the 0.5 and 1 mg/mL CMNPs were higher compared with the blank control, while none of the other groups significantly differed from the control group. As shown in Figure 6C, each MNPs group had a cell spreading area much higher than the control group. At the same time, the cell spreading area of ​​the 0.5 mg/mL MNPs group was higher than that of the 0.5 mg/mL CMNPs group. In addition, as illustrated in Figure 6D, the cells in the MNPs group were elongated by contrast with the control group, while the cell elongation rate of the CMNPs group did not differ substantially from the control group.

We used the CCK-8 assay to investigate how MNPs and CMNPs affected the viability of MC3T3-E1 cells after 12 and 24 h of cell culture. Figure 6E demonstrates that the cell viability of the CMNPs group was higher than the MNPs group. The result indicates that the modification of PL-CDs on the surface of MNPs significantly improved the biocompatibility of MNPs and that the CMNPs could promote cell proliferation. It is reasonable to deduce that excellent cytocompatibility comes from PL-CDs since CDs have been reported to have low toxicity (Lim et al., 2015). In addition, according to the research of Li et al., cationic CDs derived from lysine and arginine not only exhibited antibacterial activities but could also accelerate the proliferation process of NIH 3T3 cells (Li J. et al., 2020). One possible reason for the cell proliferation-promotion property of CMNPs could be attributed to the intracellular ROS generation through the reaction of electron-hole pairs formed by CDs and the intracellular molecular oxygen or water (Wu et al., 2014; Chong et al., 2016). The intrinsically generated ROS could activate the action of cell stress, thus stimulating cell mitosis and promoting cell proliferation. The amino functional groups on the surface of CMNPs could be another reason for the favorable cytocompatibility since amino groups could introduce local positive charge and potentially attach to negatively charged proteins and cells, thereby enhancing cell adhesion and proliferation (Zeng et al., 2018; Rathinavel et al., 2022). The above experimental results imply that CMNPs displayed excellent cytocompatibility.

**FIGURE 7:**
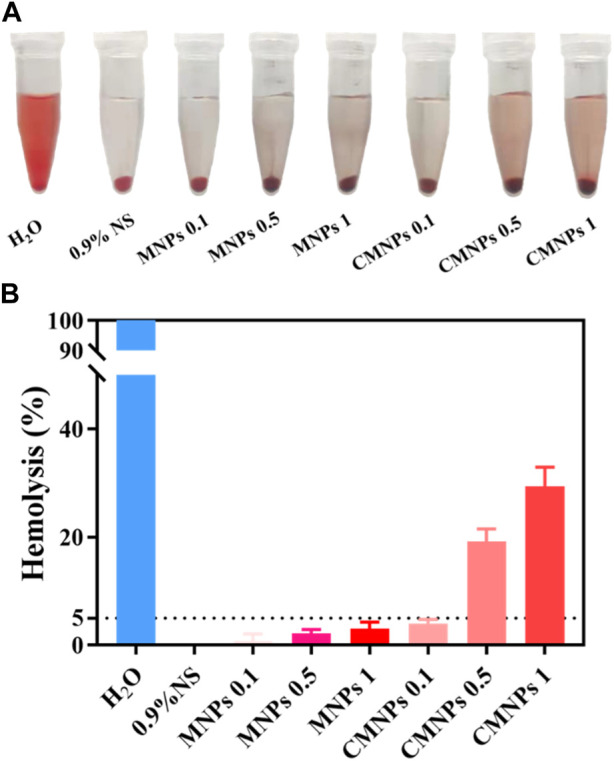
**(A)** Photographs of RBCs after MNPs and CMNPs treatment. **(B)** Hemolysis rate of erythrocytes after the treatment of samples.

In the *in vivo* application, biological materials will inevitably come into contact with blood. Therefore, the blood compatibility of biological materials is essential for their safe clinical application (Li et al., 2015; Hao et al., 2022). Hemolysis experiments were applied to evaluate the effects of MNPs and CMNPs on human red blood cells (RBCs), and Figure 7A shows photographs of RBCs after MNPs and CMNPs treatment. As shown in Figure 7B, the hemolysis rates of the 1 and 0.5 mg/ml CMNPs groups were significantly higher than those of the negative control and other experimental groups, while the hemolysis rates of MNPs groups with different concentrations and 0.1 mg/mL CMNPs were all lower than 5%. The result may be because those PL-CDs are modified with a large number of positively charged amino groups, which bind to negatively charged proteins on the surface of RBCs and cause the destruction of cell membranes, thereby significantly increasing the hemolysis rate (Kozelskaya et al., 2016). The above experiments suggest that CMNPs had good cytocompatibility, and low concentrations of CMNPs possessed acceptable hemocompatibility and could be used as potential biomedical materials for scientific research and clinical work.

## 4 Conclusion

In summary, antibacterial PL-CDs modified MNPs (CMNPs) were synthesized for the first time by linking MNPs and PL-CDs through the imine bond generated by the Schiff-base reaction. Results verified that the synthesized CMNPs had a better dispersion capacity than MNPs, and the surface modification of PL-CDs endowed CMNPs with better stability in an aqueous solution. Moreover, the positive-charged CMNPs displayed excellent antibacterial properties, though the effect was weaker than MNPs. Meanwhile, the *in vitro*biocompatibility test indicated that CMNPs exhibited satisfactory cyto/hemocompatibility. Thus, the developed CMNPs could be used as a novel antibacterial material and has great potential for clinical application.

## Data Availability

The original contributions presented in the study are included in the article/supplementary material, further inquiries can be directed to the corresponding authors.
